# The prognostic role of an optimal machine learning model based on clinical available indicators in HCC patients

**DOI:** 10.3389/fmed.2024.1431578

**Published:** 2024-07-17

**Authors:** Xiaoying Lou, Shaohui Ma, Mingyuan Ma, Yue Wu, Chengmei Xuan, Yan Sun, Yue Liang, Zongdan Wang, Hongjun Gao

**Affiliations:** ^1^Department of Clinical Laboratory, State Key Laboratory of Molecular Oncology, National Cancer Center/National Clinical Research Center for Cancer/Cancer Hospital, Chinese Academy of Medical Sciences and Peking Union Medical College, Chaoyang District, Beijing, China; ^2^Department of Statistics, Department of Electrical Engineering and Computer Sciences, University of California, Berkeley, CA, United States; ^3^Department of Clinical Laboratory, Shanxi Province Cancer Hospital/Shanxi Hospital Chinese Academy of Medical Sciences, Taiyuan, Shanxi, China

**Keywords:** hepatocellular carcinoma (HCC), overall survival (OS), OS-related clinical characteristic (OCC) panel, progression-free survival (PFS), random survival forests (RSF)

## Abstract

Although methods in diagnosis and therapy of hepatocellular carcinoma (HCC) have made significant progress in the past decades, the overall survival (OS) of liver cancer is still disappointing. Machine learning models have several advantages over traditional cox models in prognostic prediction. This study aimed at designing an optimal panel and constructing an optimal machine learning model in predicting prognosis for HCC. A total of 941 HCC patients with completed survival data and preoperative clinical chemistry and immunology indicators from two medical centers were included. The OCC panel was designed by univariate and multivariate cox regression analysis. Subsequently, cox model and machine-learning models were established and assessed for predicting OS and PFS in discovery cohort and internal validation cohort. The best OCC model was validated in the external validation cohort and analyzed in different subgroups. In discovery, internal and external validation cohort, C-indexes of our optimal OCC model were 0.871 (95% CI, 0.863–0.878), 0.692 (95% CI, 0.667–0.717) and 0.648 (95% CI, 0.630–0.667), respectively; the 2-year AUCs of OCC model were 0.939 (95% CI, 0.920–0.959), 0.738 (95% CI, 0.667–0.809) and 0.725 (95% CI, 0.643–0.808), respectively. For subgroup analysis of HCC patients with HBV, aged less than 65, cirrhosis or resection as first therapy, C-indexes of our optimal OCC model were 0.772 (95% CI, 0.752–0.792), 0.769 (95% CI, 0.750–0.789), 0.855 (95% CI, 0.846–0.864) and 0.760 (95% CI, 0.741–0.778), respectively. In general, the optimal OCC model based on RSF algorithm shows prognostic guidance value in HCC patients undergoing individualized treatment.

## 1 Introduction

Primary liver cancer is a common digestive system malignancy. According to GLOBOCAN, in 2020, the annual number of new cases of liver cancer worldwide was 905,000, ranking it sixth among cancers. The number of liver cancer deaths that year was 830,000, ranking it third globally among malignancies (1). Hepatocellular carcinoma (HCC) is the main pathological type of primary liver cancer, accounting for 85%–90% of cases. Radical resection remains a first-line treatment to promote long-term survival for patients with preserved liver function. Because the recurrence rate of HCC after hepatectomy is 50%–70%, systemic therapy and some locoregional therapies such as transarterial chemoembolization (TACE) and radiofrequency ablation are needed for advanced and metastatic disease to better control tumor progression, improve quality of life, and prolong survival (2). Surveillance and follow-up after liver cancer surgery or initial treatment are still crucial. Despite these interventions, overall survival (OS) with liver cancer remains poor (3). To individualize treatment and improve outcomes, precise prognostic indicators or panels are needed. Circulating markers are easily obtained through relatively low-cost testing and could be used to predict liver cancer survival or recurrence (4).

As an important metabolic organ, the liver is an exquisite biological factory for the synthesis of coagulation factors and metabolism-associated enzymes. Progression of HCC especially at an intermediate or advanced stage can lead to abnormal liver function and coagulation, in addition to generation of tumor markers, which partially indicate the proliferative status of the tumor. In our experience, prognosis in patients with deterioration of liver function and coagulation is less satisfactory than in those with tolerable indicators. Accumulating findings show that abnormally high alkaline phosphatase (ALP), gamma-glutamyltransferase (GGT), aspartate transaminase (AST), and lactate dehydrogenase (LDH) are associated with poor prognosis in liver cancer (5–11). Likewise, an increased coagulation indicator prothrombin time has been linked to worse progression-free survival in patients with HCC (12). For unclear reasons, a number of reports have focused on the relationship between liver cancer prognosis and the ratio of two candidate markers, such as gamma-glutamyl transpeptidase and lymphocyte count ratio, that lack any connection (13, 14). In addition, most previous studies have focused on patients with liver cancer who have undergone surgical treatment, liver transplantation, ablation, TACE, or sorafenib therapy (15–17). Moreover, many studies have had a short follow-up so that many individuals were still alive at the data cutoff, precluding accurate information about their survival period. For these reasons, the true relationship between candidate markers and OS is unclear. Meanwhile, acceptance and application of novel parameters and models in clinical practice have been slow. To date, systematic evaluations are lacking that include imaging parameters and circulating tumor and liver function markers, coagulation factors, and other laboratory tests associated with OS in HCC.

Here, we retrospectively analyzed data for patients with HCC treated at the Chinese National Cancer Center (NCC) from 2010 to 2019. The study aim was to define potential prognostic factors and develop novel models based on the panel of factors yielding optimal accuracy for assessing long-term survival in patients with HCC.

## 2 Materials and methods

### 2.1 Diagnosis of HCC

All patients were diagnosed with HCC through postoperative or biopsy pathological examination and through hematological (alpha-fetoprotein, AFP) and imaging (magnetic resonance imaging, ultrasonography, computerized tomography) studies at the National Cancer Center (NCC) from November 2010 to April 2019 and Shanxi Province Cancer Hospital (SPCH) from January 2018 to March 2022. Two independent pathologists made the histopathological diagnoses, and two independent medical laboratory technologists carried out the blood tests.

### 2.2 Data collection and inclusion and exclusion criteria

All information about diagnosis and therapy was obtained from hospital electronic medical records, and survival information was collected from our hospital’s follow-up database. Tumor diameter means the diameter of the largest tumor. Satellite nodules are defined as tumor cell nests less than 2 cm (18). All blood test values were exported from the Laboratory Information Management System. Pretherapy blood values were determined for alanine transaminase (ALT), AST, GGT, ALP, LDH, total bilirubin (TBIL), direct bilirubin (DBIL), albumin (ALB), pre-albumin (PALB), and globulin. Moreover, values for the tumor marker AFP were collected.

**FIGURE 1:**
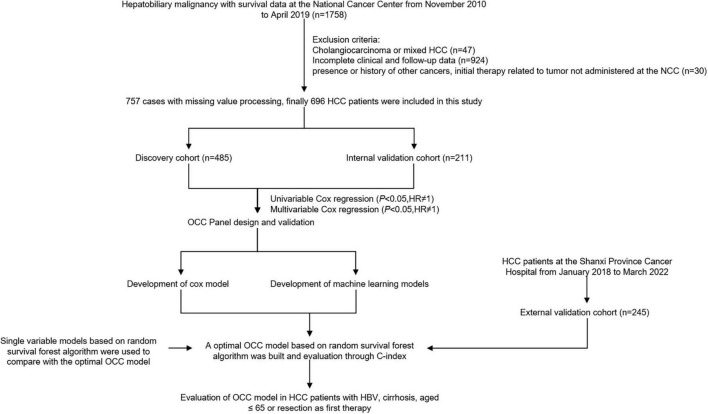
Workflow of prognostic model generation and analysis.

Inclusion criteria were as follows: (1) primary HCC diagnosis; (2) initial cancer therapy at the NCC and SPCH; (3) complete clinical case record including laboratory data; (4) complete follow-up data; and (5) cause of death relevant to the cancer. The exclusion criteria were as follows: (1) secondary liver cancer diagnosis; (2) initial therapy related to tumor not administered at the NCC and SPCH; (3) presence or history of other cancers, including other primary liver cancer such as intrahepatic cholangiocarcinoma and combined HCC and cholangiocarcinoma; and (4) death from non–cancer-related causes. OS was defined as the interval between the first date of treatment and the date of death (Figure 1).

### 2.3 Liver-related hematological markers and laboratory methods

Circulating levels of AST, ALT, ALP, GGT, creatine kinase (CK), LDH, ALB, PALB, TBIL, DBIL, β2MG (β2 microglobulin), TBA (total bile acids), TP (total protein), Transferrin, CHOL (cholesterol), HDL-CHO (high density lipoprotein cholesterol), and LDL-CHO (low density lipoprotein cholesterol) were performed using Roche Cobas c701 analyzers. AFP was measured using an electrochemiluminescence method on Roche Cobas e601 analyzers.

In summary, the average within-laboratory coefficient of variation in quality-control samples ranged between 0.907∼1.979% for ALT, 1.220∼1.670% for AST, 1.638∼1.763% for GGT, 1.756∼3.501% for ALP, 1.642∼2.230% for LDH, 1.467∼1.958% for CK, 0.755∼1.060% for TBIL, 1.892∼2.677% for DBIL, 0.978∼1.553% for TBA, 1.332∼1.406% for TP, 1.731∼1.786% for ALB, 0.936∼1.282% for PALB, 1.786∼2.317% for AFP.

### 2.4 Data preprocessing

After exclusion of variables with > 25% missing values, 8 variables were imputed using the “pmm” method in the mice R package (19). Participant data at NCC were then randomized into ‘discovery’ and ‘validation’ cohorts in an approximate 7:3 ratio.

### 2.5 Panel generation and validation

The OCC panel was established through univariate and multivariate cox regression analysis. The performance of the cox model based on OCC panel was evaluated by discrimination and calibration. The discriminative ability was assessed using the C-index and a calibration plot. Furthermore, time-dependent receiver operating characteristic (ROC) curves and corresponding area under curves (AUCs) at 1, 3, and 5 years were generated to estimate the predictive accuracy.

### 2.6 Machine-learning model development

To develop a best prognostic model based on OCC panel with high accuracy and stability performance, we performed 10 machine learning algorithms including random survival forest (RSF), elastic network (Enet), Lasso, Ridge, stepwise Cox, CoxBoost, partial least squares regression for Cox (plsRcox), supervised principal components (SuperPC), generalized boosted regression modeling (GBM), and survival support vector machine (survival-SVM). The detailed description of algorithms could be found in previous study (20).

### 2.7 Statistical analysis

Results of analyses of continuous variables with a non-normal distribution are shown as median ± interquartile range and were compared using Wilcoxon tests. Chi-square tests were used to compare categorical variables. Cox regression and Kaplan–Meier analyses were conducted using survival package. ROC curves based on the timeROC package were used to define sensitivity and specificity. The C-indices of each model were compared via CompareC package. All tests were two-sided, and unless specifically stated, P < 0.05 was considered to indicate statistical significance.

## 3 Results

### 3.1 Patient characteristics

The clinical and laboratory characteristics of patients in the discovery (507 patients) and validation (219 patients) cohorts did not significantly differ (Table 1; *P* > 0.05 for all comparisons), except for lymph node metastasis (LNM) and TBA. Average age was similar in the two groups, at 56.5 (10.6) years for the discovery cohort and 55.6 (11.1) years for the validation cohort. Most patients were male (421 [83%] in the discovery group and 183 [83.6%] in the validation group). For etiology of HCC, 598 (85.9%) HCC had hepatitis B, 54 (7.8%) had hepatitis C. Furthermore, overall, more than 619 (88.9%) HCC had cirrhosis.

**TABLE 1:** Baseline characteristics of patients with hepatocellular carcinoma in the discovery and validation cohorts.

		Discovery cohort (*n* = 507)	Validation cohort (*n* = 219)	*P*
Age (mean (SD))		56.5 (10.6)	55.6 (11.1)	0.268
Sex (%)	female	86 (17.0)	36 (16.4)	0.948
	male	421 (83.0)	183 (83.6)	
HBV (%)	No	96 (18.9)	32 (14.6)	0.195
	Yes	411 (81.1)	187 (85.4)	
HCV (%)	No	468 (92.3)	204 (93.2)	0.808
	Yes	39 (7.7)	15 (6.8)	
Cirrhosis (%)	No	80 (15.8)	27 (12.3)	0.276
	Yes	427 (84.2)	192 (87.7)	
Tumor diameter (mean (SD))		5.2 (3.3)	5.2 (3.3)	0.956
PVTT (%)	No	468 (92.3)	209 (95.4)	0.168
	Yes	39 (7.7)	10 (4.6)	
LNM (%)	No	490 (96.6)	218 (99.5)	0.041
	Yes	17 (3.4)	1 (0.5)	
Satellite nodules (%)	No	461 (90.9)	193 (88.1)	0.306
	Yes	46 (9.1)	26 (11.9)	
AFP (median [IQR])		38.3 [5.2, 751.2]	42.6 [6.0, 768.1]	0.749
ALT (median [IQR])		29.0 [21.0, 43.5]	30.0 [23.0, 43.0]	0.438
AST (median [IQR])		30.0 [22.5, 43.0]	30.0 [23.0, 41.0]	0.586
LDH (median [IQR])		173.0 [153.0, 199.5]	172.0 [152.0, 191.5]	0.209
GGT (median [IQR])		56.0 [33.0, 100.0]	53.0 [31.5, 87.0]	0.289
ALP (median [IQR])		82.0 [68.0, 103.0]	78.0 [65.5, 99.5]	0.084
CK (median [IQR])		69.0 [53.0, 100.5]	77.0 [56.0, 106.0]	0.086
TBA (median [IQR])		6.0 [3.3, 11.6]	5.2 [2.8, 9.8]	0.03
TBIL (median [IQR])		12.4 [9.5, 16.2]	12.2 [9.4, 15.8]	0.563
DBIL (median [IQR])		4.8 [3.7, 6.6]	4.6 [3.8, 6.2]	0.518
CHOL (median [IQR])		4.1 [3.6, 4.7]	4.2 [3.6, 4.7]	0.688
HDL-CHO (median [IQR])		1.2 [0.9, 1.4]	1.1 [0.9, 1.3]	0.113
LDL-CHO (median [IQR])		2.6 [2.1, 3.2]	2.7 [2.2, 3.2]	0.286
β2MG (median [IQR])		2.0 [1.8, 2.4]	2.0 [1.7, 2.4]	0.2
Transferrin (median [IQR])		234.1 [210.5, 268.3]	230.1 [204.7, 262.6]	0.077
TP (median [IQR])		70.2 [66.2, 74.4]	70.5 [66.3, 74.3]	0.872
ALB (median [IQR])		42.8 [39.6, 45.5]	43.0 [39.2, 46.0]	0.671
PALB (median [IQR])		19.0 [15.0, 23.0]	19.0 [15.0, 24.0]	0.372
First therapy (%)	Radiation	17 (3.4)	4 (1.8)	0.658
	Resection	392 (77.3)	176 (80.4)	
	RFA	22 (4.3)	9 (4.1)	
	TACE	76 (15.0)	30 (13.7)	

The average tumor diameter of the HCC patients in each group was 5.2 (3.3) cm. Eighteen (2.5%) patients had lymphatic metastasis, one of whom had more than one lymph node metastasis. Moreover, in the discovery and validation cohorts combined, 49 (7%) patients had portal vein tumor thrombosis (PVTT). In patients who underwent hepatectomy in the discovery and validation cohorts, 39 (7.7%) and 17 (7.8%) had well differentiated HCC, 261 (51.5 %) and 112 (51.1%) had moderately differentiated HCC, and 207 (40.8%) and 90 (41.1%) patients had poorly differentiated HCC. Overall, in the two cohorts combined, 72 (10.3%) of patients had satellite nodules.

For laboratory characteristics, the distribution of AFP, ALT, AST, LDH, GGT, ALP, CK, TBIL, DBIL, CHOL, HDL-CHO, LDL-CHO, β2MG, Transferrin, TP, ALB and PALB did not differ between the two groups (P > 0.05).

Regarding adjuvant treatments, 106 (15.2%) overall had TACE, 31 (4.5%) had radiofrequency ablation, and 21 (3%) had radiation therapy. More than 568 (77%) patients overall underwent liver resection.

### 3.2 Prognosis panel design and validation

**FIGURE 2:**
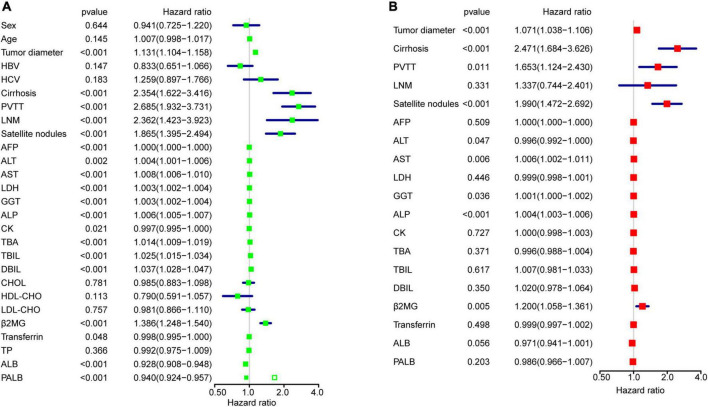
OCC panel design. **(A)** The univariable Cox regression analysis for all 27 variables in the entire cohort after imputing missing data. **(B)** The multivariable Cox regression analysis for 19 variables in the entire cohort.

To identify variables related to OS in HCC, univariable and multivariable Cox regression analysis were performed (Figure 2). Univariable analysis revealed that values for tumor diameter, cirrhosis, PVTT, LNM, satellite nodules, AFP, ALT, AST, LDH, GGT, ALP, CK, TBA, TBIL, DBIL, β2MG, Transferrin, ALB and PALB were all related to OS in HCC (Figure 2A). 17 variables associated with clinical prognosis in HCC were selected for the multivariable model analysis. The results indicated that values for tumor diameter, cirrhosis, PVTT, satellite nodules, ALT, AST, GGT, ALP and β2MG were independent prognostic factors in HCC (Figure 2B), and we defined these 9 variables as OS-related clinical characteristic (OCC) panel.

A cox model predicting OS in HCC was first constructed based on OCC panel (Supplementary Figure 1). The calibration curves demonstrated a favorable consistency between nomogram predictions and observed outcomes for 1-, 3-, and 5-year OS in the discovery and validation cohorts (Supplementary Figures 1A, B). At 1, 3, and 5 years, the AUC values in the ROC curve analysis were respectively 0.794 (95% CI, 0.744–0.844), 0.754 (95% CI, 0.710–0.798), and 0.778 (95% CI, 0.733–0.822) in the discovery cohort and 0.721 (95% CI, 0.632–0.809), 0.724 (95% CI, 0.653–0.796), and 0.747 (95% CI, 0.677–0.818) in the validation cohort (Supplementary Figures 1C, D). The C-indexes of cox model were 0.725 (95% CI, 0.710–0.740), and 0.686 (95% CI, 0.661–0.711), respectively.

These results highlighted that the OCC panel had a superior diagnostic performance for predicting prognosis of HCC.

### 3.3 Establishment of an optimal Model Based on OCC Panel for Prognosis predicting of Patients With HCC

**FIGURE 3:**
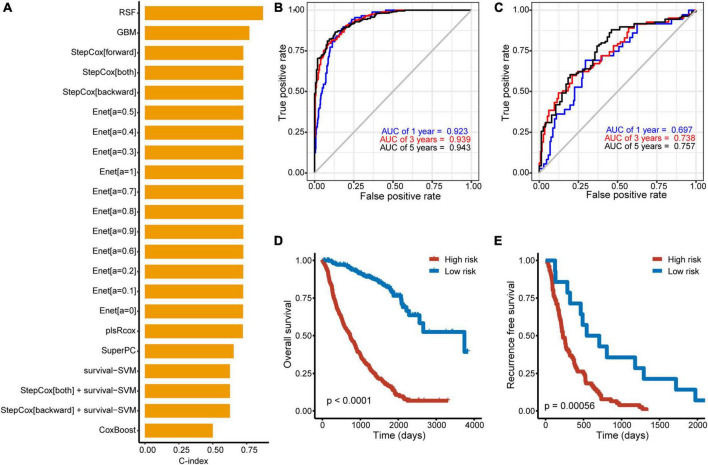
Construction of machine learning models based on OCC panel. **(A)** Prediction models based on machine learning algorithms and its calculated C-index of each model in the entire cohort. **(B, C)** The ROC curve of the OCC model based on random forest algorithm in the discovery **(B)** and validation cohort **(C)**. **(D, E)** Kaplan–Meier curves of OS according to the OCC model in the discovery **(D)** and validation cohort **(E)**.

To improve the discriminatory ability of cox model, OCC panel was used to develop different machine learning models (Figure 3). Further, the performance of each model across discovery and validation cohorts was assessed by mean C-indexes (Figure 3A). A best OCC model based on random forest algorithm was selected with a highest mean C-index of 0.781 (95% CI, 0.765-0.798) (Table 2). ROC analysis measured the discrimination of OCC model, with 1-, 3-, and 5-year AUCs of 0.923 (95% CI, 0.898–0.948), 0.940 (95% CI, 0.920–0.959), and 0.943 (95% CI, 0.923–0.963) in discovery cohort; 0.697 (95% CI, 0.603–0.790), 0.738 (95% CI, 0.667–0.810), and 0.757 (95% CI, 0.687–0.827) in validation cohort, respectively (Figures 3B, C). Furthermore, Kaplan-Meier analysis was conducted to validate the discriminative ability of OCC model. Patients with high risk had significantly worse OS and PFS relative to patients with low risk in the entire cohort (all *P* < 0.001, Figures 3D, E). To further validate the robustness of our OCC model, we validated OCC model in an external validation cohort with 245 HCC patients and the C-index was 0.648 (95% CI: 0.630–0.667, Supplementary Figure 2). ROC analysis measured the discrimination of OCC model, with 1-, 2-, and 3-year AUCs of 0.701 (95% CI, 0.636–0.767), 0.725 (95% CI, 0.643–0.808), and 0.750 (95% CI, 0.667–0.834) in the external validation cohort (Supplementary Figure 2A). Kaplan-Meier analysis demonstrated that patients with high risk had significantly worse OS relative to patients with low risk in the external validation cohort (*P* < 0.001, Supplementary Figure 2B).

**TABLE 2:** The mean C-indexes for discovery and validation cohorts in different models.

Model	C-index (95% CI)
RSF	0.781 (0.765–0.798)
GBM	0.735 (0.716–0.754)
Enet[a = 0.4]	0.714 (0.694–0.733)
Enet[a = 0.8]	0.714 (0.694–0.733)
Enet[a = 0.5]	0.714 (0.694–0.733)
Enet[a = 0.7]	0.714 (0.694–0.733)
Enet[a = 0.6]	0.714 (0.694–0.733)
Enet[a = 0.3]	0.714 (0.694–0.733)
Enet[a = 1]	0.714 (0.694–0.733)
Enet[a = 0.9]	0.714 (0.694–0.733)
StepCox[backward]	0.713 (0.694–0.733)
StepCox[both]	0.713 (0.694–0.733)
StepCox[forward]	0.713 (0.694–0.733)
Enet[a = 0.2]	0.713 (0.694–0.732)
Enet[a = 0.1]	0.713 (0.694–0.732)
Enet[a = 0]	0.712 (0.693–0.731)
plsRcox	0.711 (0.691–0.730)
SuperPC	0.641 (0.620–0.662)
StepCox[backward] + survival-SVM	0.602 (0.580–0.623)
StepCox[both] + survival-SVM	0.602 (0.580–0.623)
Survival-SVM	0.602 (0.580–0.623)
CoxBoost	0.500 (0.500–0.500)

Taken together, these results suggested that the OCC model based on random forest algorithm is superior to other machine-learning models based on OCC panel for predicting prognosis of HCC.

### 3.4 Comparison of the performance between OCC model and single predictors-based models

**FIGURE 4:**
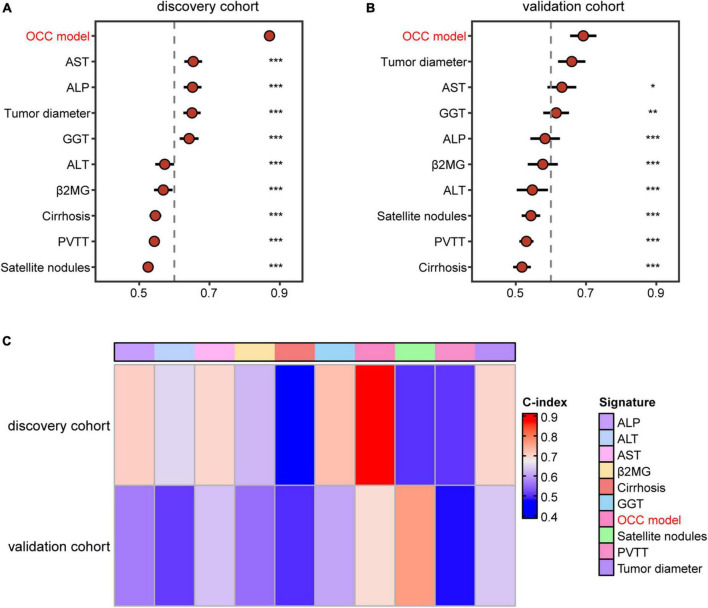
Comparison of the performance of OCC model and other single indicators in predicting OS. **(A, B)** The C-index comparison of OCC model and other clinical prognostic indicators using cox regression analysis in the training **(A)** and validation cohort **(B)**. **(C)** The C-index comparison of OCC model and other clinical prognostic indicators using random forest algorithm in the discovery and validation cohorts.

Previous studies reported that clinical characteristics like tumor diameter and clinical biomarker alterations like AST were also used to assess the prognosis of HCC in clinical practice (21, 22). Therefore, we compared the performance of OCC model with other independent prognostic indicators in predicting prognosis. As displayed in Figures 4A, B, OCC model had distinctly superior accuracy than the other single predictors-based cox models including tumor diameter, cirrhosis, PVTT, satellite nodules, ALT, AST, GGT, ALP and β2MG in both discovery and validation cohorts (all P < 0.05). In addition, we also displayed the predictive superiority of OCC model with other single predictors-based RSF models. The heatmap of C-index of OCC model and other clinical prognostic indicators demonstrated that OCC model always achieved the highest mean C-index in both discovery and validation cohorts (Figure 4C). These results suggested that the OCC model is superior to single predictors-based models for predicting prognosis of HCC.

### 3.5 The optimized OCC model performs robustly in predicting prognosis of HCC patients with HBV, aged less than 65, cirrhosis or resection as first therapy

**FIGURE 5:**
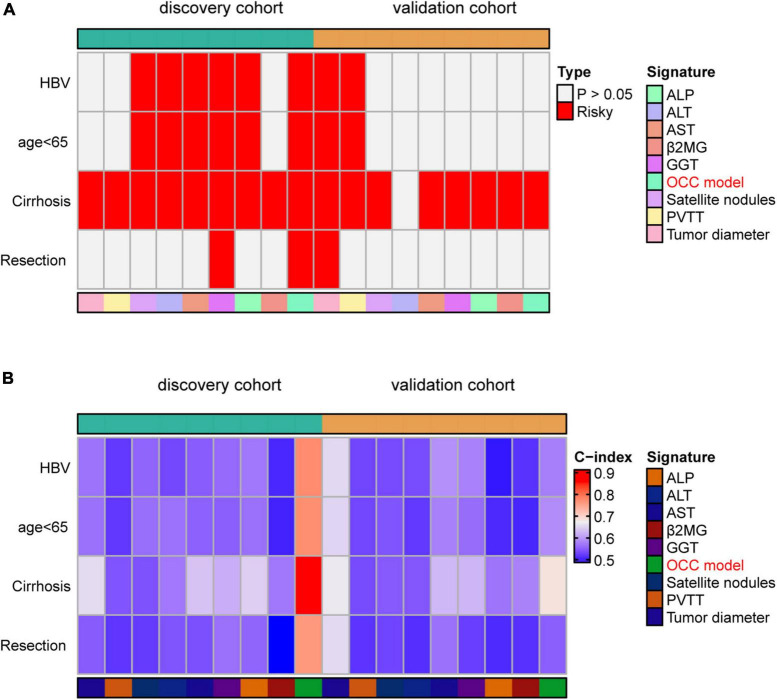
Performance of OCC model in HCC patients with HBV, aged less than 65, cirrhosis or resection as first therapy. **(A)** The heatmap of univariate cox regression analysis of OCC model for HCC patients with HBV, aged less than 65, cirrhosis or resection as first therapy in discovery and validation cohort. **(B)** The heatmap of C-index of OCC model for HCC patients with HBV, aged less than 65, cirrhosis or resection as first therapy in discovery and validation cohort.

Since our HCC patients 88.9% had cirrhosis and 85.9% had HBV, the subgroup analysis was meaningful for the entire cohort. Thus, prognosis model may be more applicable to patients with HBV, cirrhosis and so on. To assess whether our OCC model had the same or similar prognostic value in different populations, we used OCC model to predict survival of HCC patients with HBV, aged less than 65, cirrhosis or resection as first therapy. Univariate cox regression was performed in four populations for each indicator and observed that OCC model was positively associated with bad prognosis in both two cohorts in HCC patients with cirrhosis (Figure 5A). However, OCC model didn’t have consistent prognostic role in both two cohorts in HCC patients with aged less than 65, HBV or resection as first therapy. The heatmap of C-index of OCC model and other clinical prognostic indicators demonstrated that OCC model always achieved the highest C-index in discovery cohort for four subgroups (Figure 5B), especially in HCC patients with cirrhosis. In addition, the OCC model also achieved the highest AUC in HCC patients with cirrhosis (Table 3). Collectively, these results showed that OCC model provided a robust accuracy in predicting survival of HCC patients with HBV, aged less than 65, cirrhosis or resection as first therapy.

**TABLE 3:** The AUCs of OCC model for predicting OS in different subgroups.

Cohort	HBV		Age < 65		Cirrhosis		Resection	
	Discovery cohort	Validation cohort	Discovery cohort	Validation cohort	Discovery cohort	Validation cohort	Discovery cohort	Validation cohort
1 years AUC	0.852	0.57	0.854	0.590	0.913	0.690	0.854	0.546
3 years AUC	0.876	0.559	0.887	0.582	0.930	0.730	0.851	0.516
5 years AUC	0.924	0.651	0.883	0.643	0.939	0.754	0.837	0.577

## 4 Discussion

To improve outcomes as much as possible, all of the patients with HCC in this study were treated with at least two of the following therapies: hepatic resection, liver TACE, radiofrequency ablation, and radiotherapy. Most patients were treated with TACE up to 10 times or with all of these therapies. Nevertheless, only one patient in the overall cohort ( < 0.25%) had a survival period > 10 years, and only 26 patients (6.5% of overall cohort) had a survival of > 5 years. Thus, the identification and screening of prognostic indicators and models have obvious clinical significance in assisting individualized treatment of patients with liver cancer by highlighting targets for therapeutic focus. As routine surveillance indicators, enzymes associated with liver function in the peripheral blood and imaging exams, such as ultrasound, are usually simple and accessible.

In this study, we performed univariable and multivariable cox regression analysis and found that tumor diameter, cirrhosis, PVTT, satellite nodules, ALT, AST, GGT, ALP and β2MG were independent prognostic factors. Subsequently, we defined these 9 markers as OCC panel and validation its prognostic value by cox regression analysis. Furthermore, different machine learning models were constructed to improve the discriminatory ability of OCC panel. Overall, the C-indexes and AUCs indicated an optimal discriminative ability of the random forest model based on OCC panel compared with other models based on single factors or OCC panel, suggesting that the described OCC model is a reliable model for evaluating prognosis.

Predictive performance was obviously improved through constructing models with tumor diameter, cirrhosis, PVTT, satellite nodules, ALT, AST, GGT, ALP and β2MG. Regarding tumor diameter, Michael et al. studied a large group of patients with different background liver diseases and found that tumor size alone was a limited prognostic factor (23). Our cox analysis showed that tumor diameter was an independent prognostic factor, but combined with other indicators, the prognostic performance would be better. This suggests that tumor size plays an important role in predicting the prognosis of patients. Cirrhosis was traditionally considered as cause of HCC (24), and cirrhosis was also an independent prognostic indicator. PVTT is common in patients with HCC, and those who develop PVTT usually have an aggressive disease course, decreased liver function reserve, limited treatment options, higher recurrence rates after treatment, and worse OS (25). In the single and multiple Cox regression analyses in the current work, patients with PVTT had a poor prognosis, confirming this association. Satellite nodules is a well-known risk factor for HCC, which usually cannot be detected on imaging modalities (26). Using our OCC panel, satellite nodules help to improve the prognostic stratification for HCC patients. Among 5 laboratory parameters, ALP is essential laboratory tests in the National Comprehensive Cancer Network guidelines for the treatment of liver cancer. Serum ALP generally arises from liver, kidney, and bone, so that its elevation does not necessarily trace solely to the liver. However, the source can be determined by combining multiple indicators and further analysis of ALP isozymes (27). Low ALP can indicate a healthy liver, suggesting why lower ALP values correlated with better prognosis in our cohorts. ALT, AST and GGT were related to liver functional impairment which might be caused by various diseases such as liver fibrosis and cirrhosis (28, 29). β2MG is a low molecular weight protein which is produced by lymphocytes, platelets, and multinucleated leukocytes, and was associated with prognosis of brain injury (30). Our cox regression analysis discovered the novel prognostic role of β2MG in HCC.

In this study, we used both traditional Cox regressions and several machine-learning algorithms to accurately predict survival time in HCC. Several studies have examined the significance of machine-learning techniques in cancer prognosis (31–34). When comparing accuracy among models in predicting OS in HCC patients, the RSF model was the best with highest mean C-index of 0.781 (95% CI, 0.765-0.798) in NCC cohort. Moreover, OCC model had the same or similar prognostic value in HCC patients with HBV, aged less than 65, cirrhosis or resection as first therapy. This survival-time–dependent prediction ability has been reported in other studies (35, 36), and different parameter combinations may have different prediction effects in patients with different prognoses. The advantage of our machine-learning model is that it allowed for precise grouping of smaller subsets of patients to classify subgroups more accurately with similar prognoses.

Our study has several strengths. Our study provides non-loss follow-up data of liver cancer patients receiving different treatments. This data is helpful for doctors to predict the prognosis of patients in the real world through easily available indicators, so as to personalize treatment and management. Secondly, our machine learning model was superior to conventional cox regression methods by assessing model performance such as both C-indexes and AUCs. In addition, the multiple variables-based panel was superior to single variable like tumor diameter when comparing RSF models based on both OCC panel and single OS-related indicators. Most similar investigations have constructed prognosis models for HCC patients undergoing monotherapy or limited treatment, such as liver resection, TACE, or chemotherapy (37–41). Inclusion of patients in our study was not limited based on specific therapy, so our models are more broadly generalizable in clinical practice.

Histopathological parameters such as macrovascular invasion or tumor differentiation may increase prognostic accuracy for HCC patients (42–44). However, < 40% of patients in the current cohort were unable to undergo resection and had no histopathological information available because of the advanced stage of the disease at diagnosis. Therefore, the application of histopathological parameters is extremely limited in constructing prognostic models. Ultrasound and enhanced computed tomography are crucial for clinical diagnosis of liver cancer, providing information about PVTT status and tumor size for the clinical evaluation of patients with HCC. The prognostic panel constructed in this study consisted of laboratory and imaging examinations for HCC patients with or without pathological diagnosis. For these reasons, we believe that our models can be easily developed in the future.

Our study also has several limitations. Firstly, it is a retrospective study with selection bias, and the results require further validation in larger population. However, this bias has been minimized through two independent cohorts. Secondly, the complexity of our RSF algorithm-based prognosis model hindered its application in clinical practice, but our available and repeatable codes avoided this problem.

## 5 Conclusion

In conclusion, we systematically evaluated the prognostic value of clinical laboratory and pathologic variables and constructed OCC signatures for predicting survival of HCC patients using machine learning methods. This study contributes to understanding of abnormal clinical characteristics in HCC and provides additional insight into risk stratification for these patients.

## Data availability statement

The raw data supporting the conclusions of this article will be made available by the authors, without undue reservation.

## Ethics statement

The studies involving humans were approved by the study is original and is not under consideration for publication in another journal. The study has been approved by the Medical Research Centre (MRC) at the National Cancer Center, China. All methods were performed in accordance with the relevant guidelines and regulations and according to the principles laid down in the Declaration of Helsinki. All the authors reviewed and approved the final manuscript. Due to the nature of this retrospective study and the preserved anonymity of patients, a waiver of informed consent was obtained from the Medical Research Centre (MRC) at the National Cancer Center, China. The studies were conducted in accordance with the local legislation and institutional requirements. Written informed consent for participation in this study was provided by the participants’ legal guardians/next of kin.

## Author contributions

XL: Writing−review and editing, Data curation, Formal analysis. MM: Investigation, Software, Writing−review and editing. SM: Data curation, Software, Writing−review and editing. YW: Methodology, Writing−review and editing. CX: Data curation, Software, Writing−review and editing. YS: Data curation, Software, Writing−review and editing. YL: Writing−review & editing. ZW: Software, Investigation, Writing−review and editing. HG: Conceptualization, Validation, Writing−original draft, Writing−review and editing.
