# Supplementary figures and images for: The prognostic role of an optimal machine learning model based on clinical available indicators in HCC patients
Source: Front Med (Lausanne). 2024 Jul 17;11:1431578. doi: 10.3389/fmed.2024.1431578 (PMC11288914; doi:10.3389/fmed.2024.1431578)

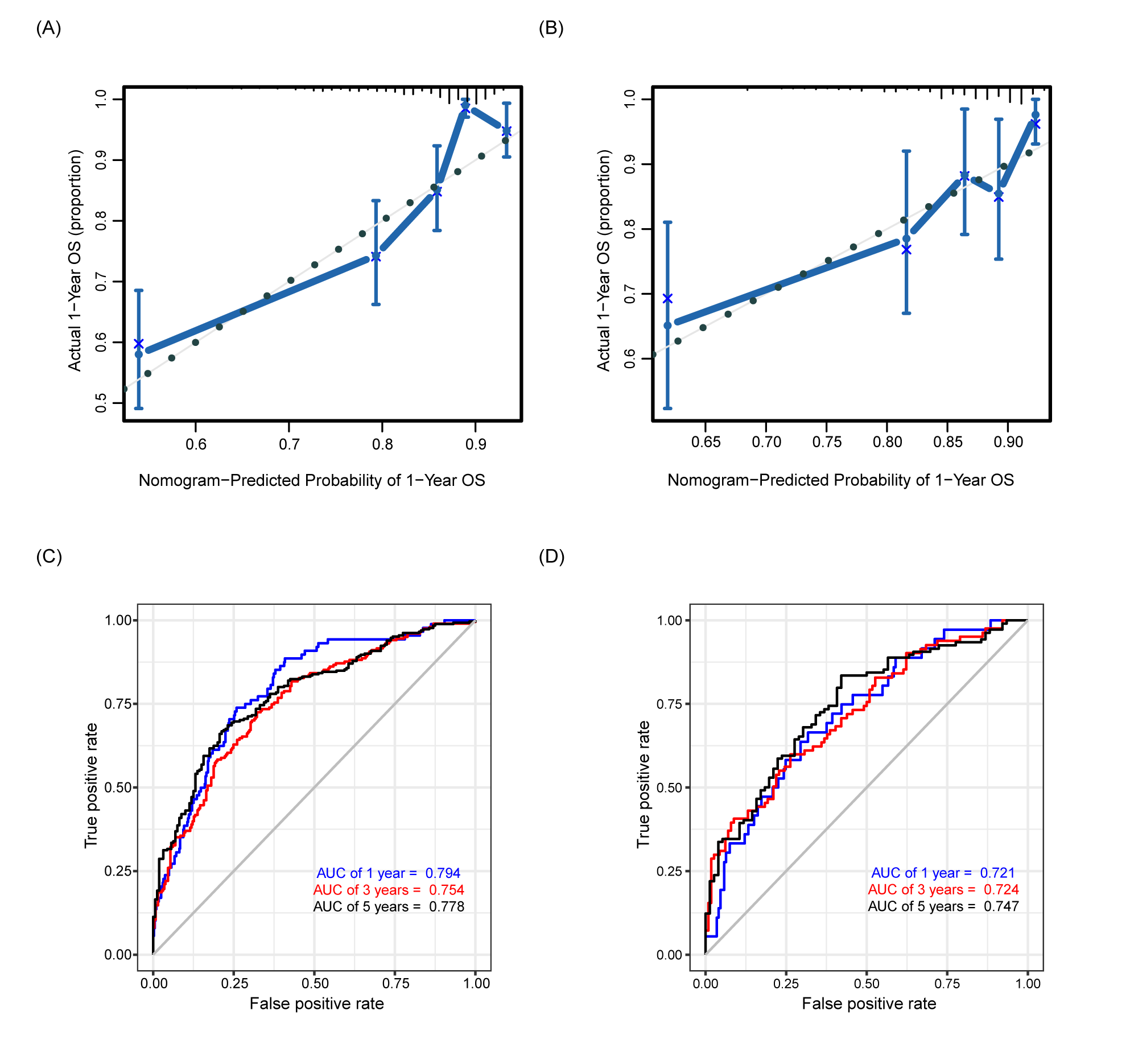

Supplement: Supplementary file 1 [file Image_1.TIF]

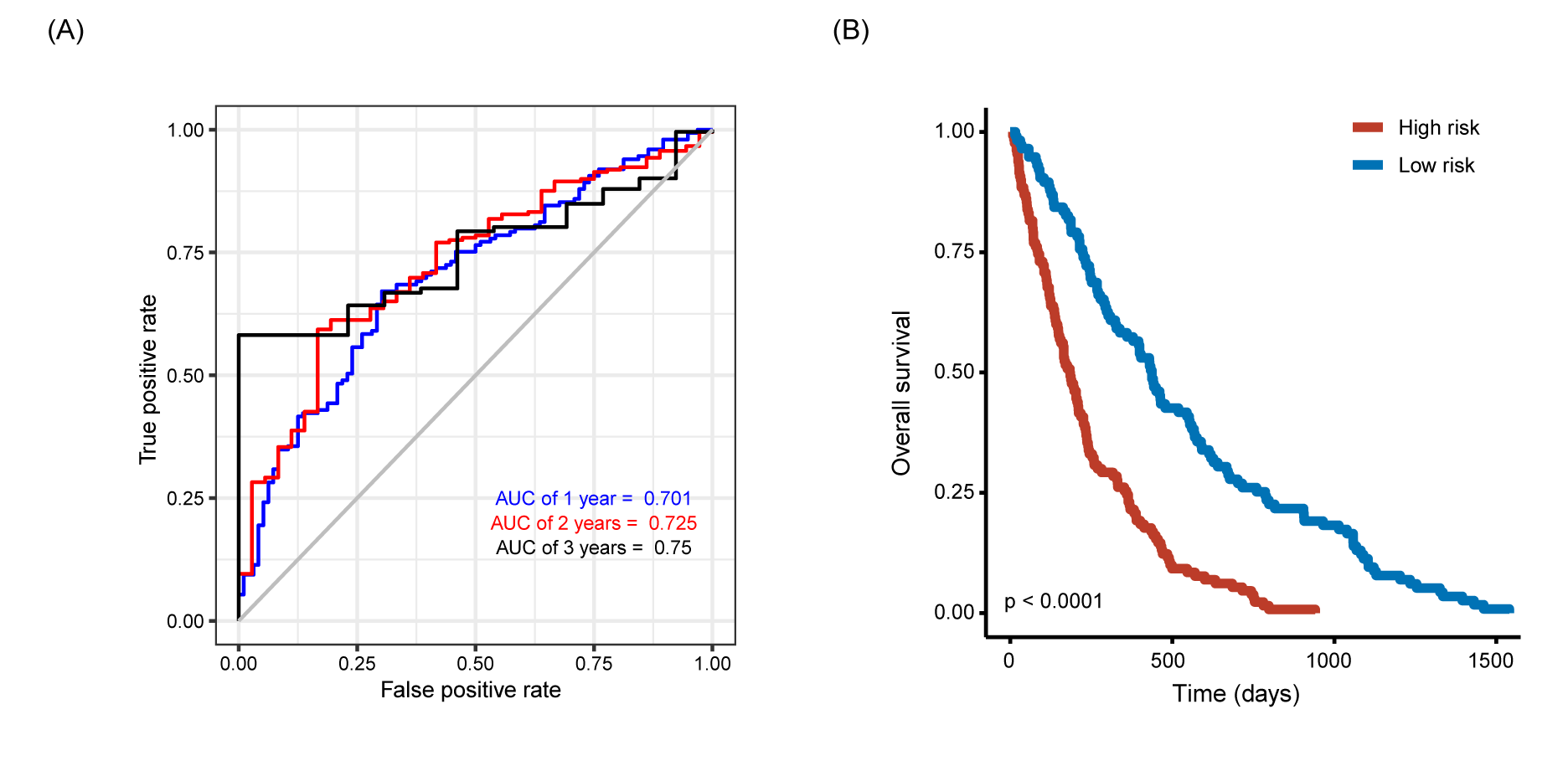

Supplement: Supplementary file 2 [file Image_2.TIF]
